# Supplementary material for: Tead1a Initiates Transcriptional Priming Through the TEAD1a/YAP‐Notch1‐Spi1/Cebpα Axis to Promote Neutrophil Fate
Source: Adv Sci (Weinh). 2025 Aug 28;12(41):e05441. doi: 10.1002/advs.202505441 (PMC12591127; doi:10.1002/advs.202505441)
Supplement: Supplementary file 4 — Supplementary Dataset [file ADVS-12-e05441-s004.zip › 3-8.zip!/3-8/0904/0904/06.celltype_ratio/ratio_celltype_sample_facet.pdf]

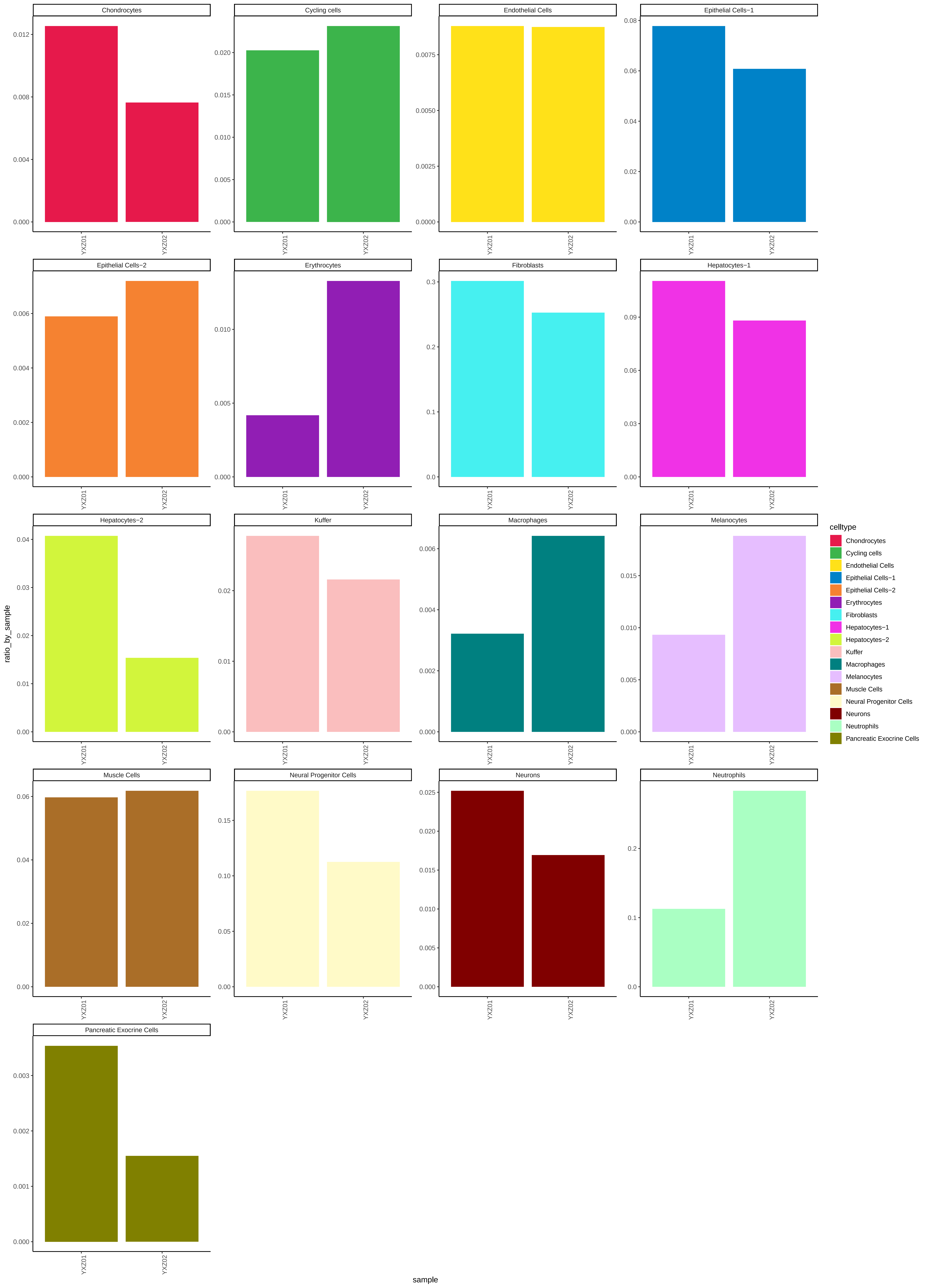

Chondrocytes
Cycling cells
Endothelial Cells
Epithelial Cells−1
Epithelial Cells−2
Erythrocytes
Fibroblasts
Hepatocytes−1
Hepatocytes−2
Kuffer
Macrophages
Melanocytes
Muscle Cells
Neural Progenitor Cells
Neurons
Neutrophils
Pancreatic Exocrine Cells
YXZ01
YXZ02
ratio_by_sample
sample
celltype
Chondrocytes
Cycling cells
Endothelial Cells
Epithelial Cells−1
Epithelial Cells−2
Erythrocytes
Fibroblasts
Hepatocytes−1
Hepatocytes−2
Kuffer
Macrophages
Melanocytes
Muscle Cells
Neural Progenitor Cells
Neurons
Neutrophils
Pancreatic Exocrine Cells
